# Complications and Health-Related Quality of Life in Children with Various Etiologies of Early-Onset Scoliosis Treated with Magnetically Controlled Growing Rods—A Multicenter Study

**DOI:** 10.3390/jcm13144068

**Published:** 2024-07-11

**Authors:** Pawel Glowka, Pawel Grabala, Munish C. Gupta, Daniel E. Pereira, Michal Latalski, Anna Danielewicz, Michal Grabala, Marek Tomaszewski, Tomasz Kotwicki

**Affiliations:** 1 Department of Spine Disorders and Pediatric Orthopaedics, Poznań University of Medical Sciences, 28 Czerwca 1956 r. Street, no. 135/147, 61-545 Poznań, Poland; pawel.glowka@ump.edu.pl (P.G.); mtomaszewski@ump.edu.pl (M.T.); kotwicki@ump.edu.pl (T.K.); 2 Department of Pediatric Orthopedic Surgery and Traumatology, University Children’s Hospital, Waszyngtona 17, 15-274 Bialystok, Poland; 3 Paley European Institute, Al. Rzeczypospolitej 1, 02-972 Warsaw, Poland; 4 Department of Orthopaedic Surgery, Washington University in St. Louis, 660 S Euclid Ave., St. Louis, MO 63110, USA; munishgupta@wustl.edu (M.C.G.); d.e.pereira@wustl.edu (D.E.P.); 5 Paediatric Orthopaedic Department, Medical University of Lublin, Gebali 6, 20-093 Lublin, Poland; michallatalski@umlub.pl (M.L.); anna.danielewicz@umlub.pl (A.D.); 6 2nd Clinical Department of General and Gastroenterogical Surgery, The Medical University of Bialystok Clinical Hospital, Medical University of Bialystok, M. Skłodowskiej-Curie 24a, 15-276 Bialystok, Poland; michal@grabala.pl

**Keywords:** early-onset scoliosis (EOS), magnetically controlled growing rods (MCGR), pediatric spinal deformity, growing rods, juvenile scoliosis

## Abstract

**Background:** Early-onset scoliosis (EOS) refers to spinal deformities that develop and are diagnosed before the age of 10. The most important goals of the surgical treatment of EOS are to stop the progression of curvature, achieve the best possible correction, preserve motion, and facilitate spinal growth. The objectives of this multicenter study were to analyze the risk of complications among patients with EOS treated using magnetically controlled growing rods (MCGRs) and assess the patients’ and their parents’ quality of life after diagnosis and treatment with a minimum two-year follow-up. **Methods:** Patients given an ineffective nonoperative treatment qualified for surgery with MCGRs. This study involved 161 patients (90 females and 71 males) who were classified according to the etiology of curvature. The intraoperative and postoperative complications and those that occurred during the continuation of treatment with MCGRs were recorded and analyzed. The 24-item Early-Onset Scoliosis Questionnaire (EOSQ-24) was used to evaluate the patients’ quality of life and satisfaction with the treatment. **Results:** Implant-related complications requiring instrumentation revision were recorded in 26% of the patients. Medical complications occurred in 45% of the population. The EOSQ-24 revealed a significant improvement in the average scores during the follow-up. **Conclusions:** The treatment of early-onset scoliosis with MCGRs carries 66% risks of incurring medical and mechanical complications, the latter 26% of patients requiring revision procedures. Children with neuromuscular scoliosis, females, and with curvature greater than 90 degrees are at a higher risk of developing complications. Limiting the number of elective surgeries necessitated to prolong the instrumentation and treatment process for patients with MCGRs can greatly enhance their quality of life and satisfaction throughout the follow-up period.

## 1. Introduction

Early-onset scoliosis (EOS) poses a significant challenge within the field of pediatric orthopedics. EOS refers to spinal deformities that develop and are diagnosed before the age of 10 [1]. Surgical treatment is considered when nonoperative treatments, such as exercises and braces, fail. The most important goals of the surgical treatment of EOS are to stop the progression of curvature, achieve the best possible correction, preserve motion, and facilitate spinal growth and lung development [2]. The biggest disadvantage of traditional growing rods (TGRs) is the necessity of repeating surgeries every 6 to 12 months until skeletal maturity is reached [3,4,5,6,7,8]. Having multiple surgical procedures raises the likelihood of experiencing complications [4,9,10,11,12,13,14,15]. In the last decade, magnetically controlled growing rods (MCGRs) have been introduced in the treatment of EOS [9,16,17,18,19]. The aim of MCGRs is to control curve progression during growth until the spine has fully developed and fused. MCGRs limit the number of surgeries that have to be performed on patients. Magnetically driven linear actuators allow for non-invasive ambulatory lengthening [16,17,18,19,20,21]. The utilization of MCGRs for managing EOS aims to decrease the frequency of surgical interventions, leading to a potential decrease in the occurrence of complications [9,16,17,22,23,24,25,26,27]. Some other important goals of the treatment include the patients’ satisfaction, short recovery time, and early return to social activity [23,28,29,30,31,32]. The complication rate of TGRs is well defined in the literature [3,8,9,33,34,35]. The aim of this study is to define the type and the prevalence of complications related to MCGRs.

## 2. Materials and Methods

### 2.1. Setting and Patients

This successive study encompassed all the individuals who received treatment with MCGRs (MAGnetic Expansion Control; NuVasive, San Diego, CA, USA) for early-onset scoliosis at five different institutions spanning from 2016 to 2022. Patient identification was carried out through the examination of local surgical archives. The funding for the treatment of all the patients was entirely covered by the public healthcare system. The execution of this investigation adhered to the principles outlined in the Declaration of Helsinki (2013 revision). Following endorsement from a regional Bioethics Committee, the medical records of pediatric patients with scoliosis who underwent treatment were comprehensively reviewed. Our analysis was retrospective and focused on the patients with EOS treated using MCGRs, with a minimum follow-up period of 2 years, except in the cases where revision surgery was necessary. The patients who had undergone previous spinal procedures involving instruments other than MCGRs were not included (if revision was necessary earlier than 2 years after MCGRs implantation, such cases were also included in the study). A total of 161 patients satisfied the inclusion criteria, comprising 302 MCGRs inserted throughout the treatment process. The patients who underwent surgical intervention for spinal deformities of various causes during growth were categorized based on the EOS classification [36].

### 2.2. Outcome Parameters

Preceding the intervention, every patient underwent magnetic resonance imaging of the complete spinal column to rule out alternative pathologies affecting the spinal cord and spine. Radiological measurements were obtained prior to the commencement of surgical intervention, before the introduction of magnetic rods, immediately post-placement, and throughout the follow-up period. The Cobb angles of the major curvature (MC), thoracic kyphosis (TK), and lumbar lordosis (LL), as well as the T1–T12 and T1–S1 heights, were evaluated before, after, and during each follow-up using the methodology outlined by Cheung et al. [16,37]. The correction of the MC angle post-surgery and during monitoring (measured with the Cobb method) was computed as the ratio of the preoperative MC angle subtracted from the postoperative MC value in the coronal planes, divided by the initial MC measurement. Proximal junction kyphosis (PJK) was identified radiographically as a kyphosis angle change greater than 10° above the top two vertebrae levels compared to the initial upright postoperative image. All atypical back pain before surgery and during follow-up also was noted [38]. The documentation included data on the instrumentation; anchor type (a pedicle screw or a hook); and the occurrence of complications, namely infections, anchor pull-outs, rod breakages, pin fractures, distraction failures, adding on, and PJK. Rod replacements were also noted. Revision procedures and definitive surgeries conducted earlier than planned due to complications were categorized as unplanned [39,40,41,42,43,44].

### 2.3. Surgical Technique and Postoperative Use of MCGRs

Our surgical technique presents a less-invasive spinal procedure involving two concise incisions positioned at the superior and inferior aspects of the instrumented spine. Segmental screws or hooks, or hybrid construct screws and hooks were attached to the superior part, with segmental screws also placed on the inferior part, alongside the subfascial insertion of MCGRs, following the techniques outlined in other studies [45]. The initial MCGRs placement and correction of scoliosis were performed under neuromonitoring control in accordance with generally accepted standards described in the literature [46]. The postoperative protocol for MCGRs involved distraction, mirroring similar approaches that have been documented in the existing literature [47,48,49], albeit tailored to suit our specific patient population. A brace was worn by all the patients for 3 months, following which lengthening commenced using an external remote controller (ERC). Distraction procedures were typically conducted every 8 weeks for most patients, with the MCGRs being elongated by 2–2.5 mm depending on their individual growth potential. Regular monitoring was performed every 6 months using X-rays, or sooner if the patients reported new symptoms, such as back pain, implant prominence, or neurological issues, as well as in the cases where potential implant loosening or rod fractures were suspected during physical examination. The X-ray evaluations focused on identifying the implant fractures, assessing the screw stability, determining the rod extension length, evaluating the spinal growth parameters, and examining the curvature parameters in both the sagittal and coronal planes [45,47].

### 2.4. Complications and Health-Related Quality of Life (HRQoL)

From the categorizations of complications arising from spinal surgery in pediatric and adult populations as documented in the existing literature, we tailored and adjusted them to suit our specific requirements [42,43,44]. The subsequent definitions were employed for this investigation: adverse events and complications were categorized into intraoperative (manifested between the initial surgery and 30 days post-procedure), early postoperative (manifested between 30 days and 3 months after the procedure), or late incidents (manifested more than 3 months after the initial procedure). The various complications observed were classified into different categories: wound-related (including dehiscence, superficial wound infection, or deep surgical site infection necessitating irrigation and debridement); implant-related (such as malplacement of the implant, pull-out, rod fracture, failure of growing rod connector, or distraction failure); alignment-related (involving junctional kyphosis requiring re-instrumentation); neurologic (such as neurologic deficit or loss of intraoperative neuromonitoring signals); and other (such as dural tear, respiratory or gastrointestinal issues, or cardiopulmonary complications) [5,44,48,49,50]. Certain specific complications were only identifiable during revision surgery or conversion to Posterior Spinal Fusion (PSF), such as spontaneous fusion, rib fusion, and metallosis [15,24,25,26,51].

The patients were considered suitable candidates for undergoing a second surgery if they experienced a negative event or issue that could not be resolved with conservative methods, posed a danger to their safety and health, and required immediate surgical treatment. Those patients who received treatment with MCGRs and encountered issues like rod breakage or PJK development, or had reached full skeletal maturity, or met specific criteria for conversion to PSF between T1–T12 and T1–S1, opted for elective surgery. This study focused solely on documenting the number of complications and revision rates, as all other unexpected complications and surgeries have been thoroughly examined in previous research efforts.

The 24-item Early-Onset Scoliosis Questionnaire (EOSQ-24) is an instrument designed to assess the HRQoL specifically for children suffering from EOS, as well as their families [30,52,53]. In order to gather information, patients, along with their parents, were requested to fill out the EOSQ-24 both before the surgical procedure and during the last follow-up appointment [52,53]. The questionnaire is comprised of 24 items distributed among 11 subdomains which include general health, pain, respiratory function, mobility, body function, daily life, fatigue, emotional development, parental burden, financial impact, and satisfaction. The three main domains under consideration were quality of life, parental burden, and patients’ satisfaction. The evaluation conducted by parents and/or caregivers took into account the relevance of each item to their child’s health status (relevance), as well as the clarity of the items in terms of comprehensibility. The primary goal of treating EOS is not only to enhance the natural progression of the condition but also to enhance the health-related quality of life for children, while simultaneously reducing the burden on caregivers [17,52]. Challenges arise when attempting to measure quality of life due to the diverse nature of the population, and it should be noted that health status indicators for adults may not be directly applicable to children. In response to this issue, the EOSQ-24 was developed utilizing a psychometric approach tailored to this specific population, aiming to address these challenges comprehensively. Item values ranging from 1 to 5 were allocated to correspond with the response choices varying from poor to excellent. Subsequently, the algebraic means of the items falling under each domain were meticulously formulated and then incorporated into the equation aimed at converting raw scores into scale scores. It is anticipated that these newly derived scale scores, as a result of the transformation process, will fall within the range of 0 to 100 [31,52,53].

### 2.5. Statistical Analysis

IBM Corp.‘s SPSS Statistics version 27 was utilized for the execution of all statistical analyses within the study. Before delving into the analysis phase, a thorough examination of the data was conducted to ensure normality. This preliminary assessment involved the scrutiny of skewness and kurtosis Z-values, as well as the inspection of histograms and Q-Q plots. Furthermore, the evaluation of normal distribution was carried out through the application of the Shapiro–Wilk test, a commonly used statistical method for such assessments. The quantitative variables within the dataset underwent scrutiny through Student’s *t*-test and ANOVA, both widely recognized methods in statistical analysis for comparing means across different groups. Conversely, the qualitative variables were subjected to analysis using the chi-square test, a statistical tool commonly employed for assessing relationships between categorical variables. Moreover, Pearson’s correlation coefficient was utilized to gauge the association between the radiographic follow-up time and the heights of T1–T12 and T1–S1. The presentation of data in the study followed the convention of mean ± standard deviation unless otherwise specified. In the absence of a predetermined sample size calculation, a post-hoc power analysis was conducted for multiple linear regression, revealing a study power exceeding 0.8. To ascertain the significance of findings, a threshold of *p* < 0.05 was set for all statistical analyses, ensuring a robust evaluation of the study outcomes.

## 3. Results

### 3.1. Patients’ Characteristics

Among the cohort of 161 individuals, consisting of 90 females and 71 males, the distribution of scoliosis etiologies was as follows: 51 cases (32%) were classified as neuromuscular (NS), 42 cases (26%) as syndromic (SS), 58 cases (36%) as idiopathic (IS), and 10 cases (6%) as congenital (CS) [45,47]. In terms of age distribution, 73 patients (45%) were below 6 years old, while the remaining 88 patients (55%) were over 6 years old. The average duration of follow-up for these patients was recorded at 32.8 months, with a range spanning from 12 to 68 months. The mean age at which the patients received magnetically controlled growing rods (MCGRs) was calculated to be 7.08 years, with an age range of 2.5 to 14 years. Upon the final follow-up post MCGR removal and fusion surgery, the mean age was found to be 14.5 years, ranging from 11 to 16 years. As for the specifics of the spinal rods utilized, 66 rods (22%) with diameters of 4.5 and 5.0 mm were implanted, while the remaining 236 rods (78%) had larger diameters of 5.5 and 6.0 mm. Among the rods, 114 (38%) were of 70 mm length, with the remaining 188 rods (62%) measuring 90 mm. Treatment approaches varied, with 20 patients (12.5%) receiving single-rod constructs, and the remaining 141 patients (87.5%) undergoing treatment with double-rod constructs.

Among the cohort, 66 patients (41%) exhibited severe scoliosis exceeding 90°, while 95 patients (59%) had a primary curvature of less than 90°. Preoperative Halo Gravity Traction (HGT) was administered to 32 patients (20%), and anterior release was performed on 16 patients (10%). Those who underwent anterior release did not receive preoperative HGT. Only 73% (117 children) of all the patients in this cohort study completed the EOSQ-24 questionnaire for analysis before the surgical treatment with MCGRs and at the last follow-up.

All the radiological outcomes of this cohort were analyzed and have been published in our other studies [45,47].

### 3.2. Analysis of the Subgroups Complications

During the initial surgery, we noted 20.5% complications classified as intraoperative, which 4.3% in total needed revision surgery. All the noted complications are presented in Table 1.

We also noted several complications categorized as early postoperative, which developed between 30 days and 3 months after the procedure. These complications are listed in Table 2.

**Figure 1:**
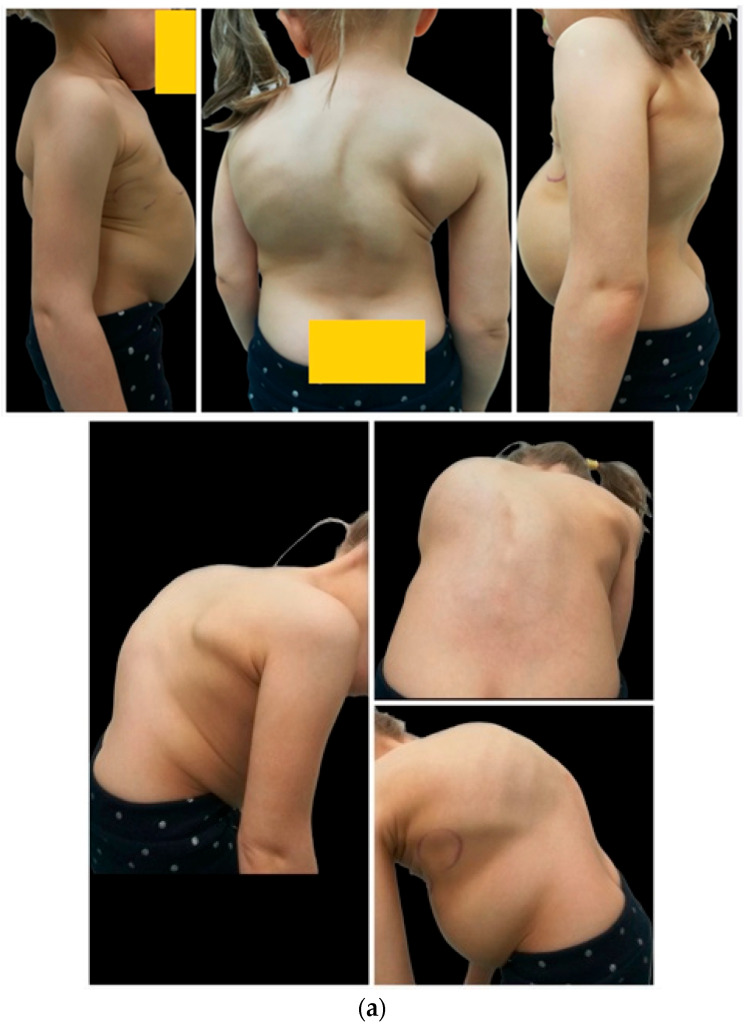

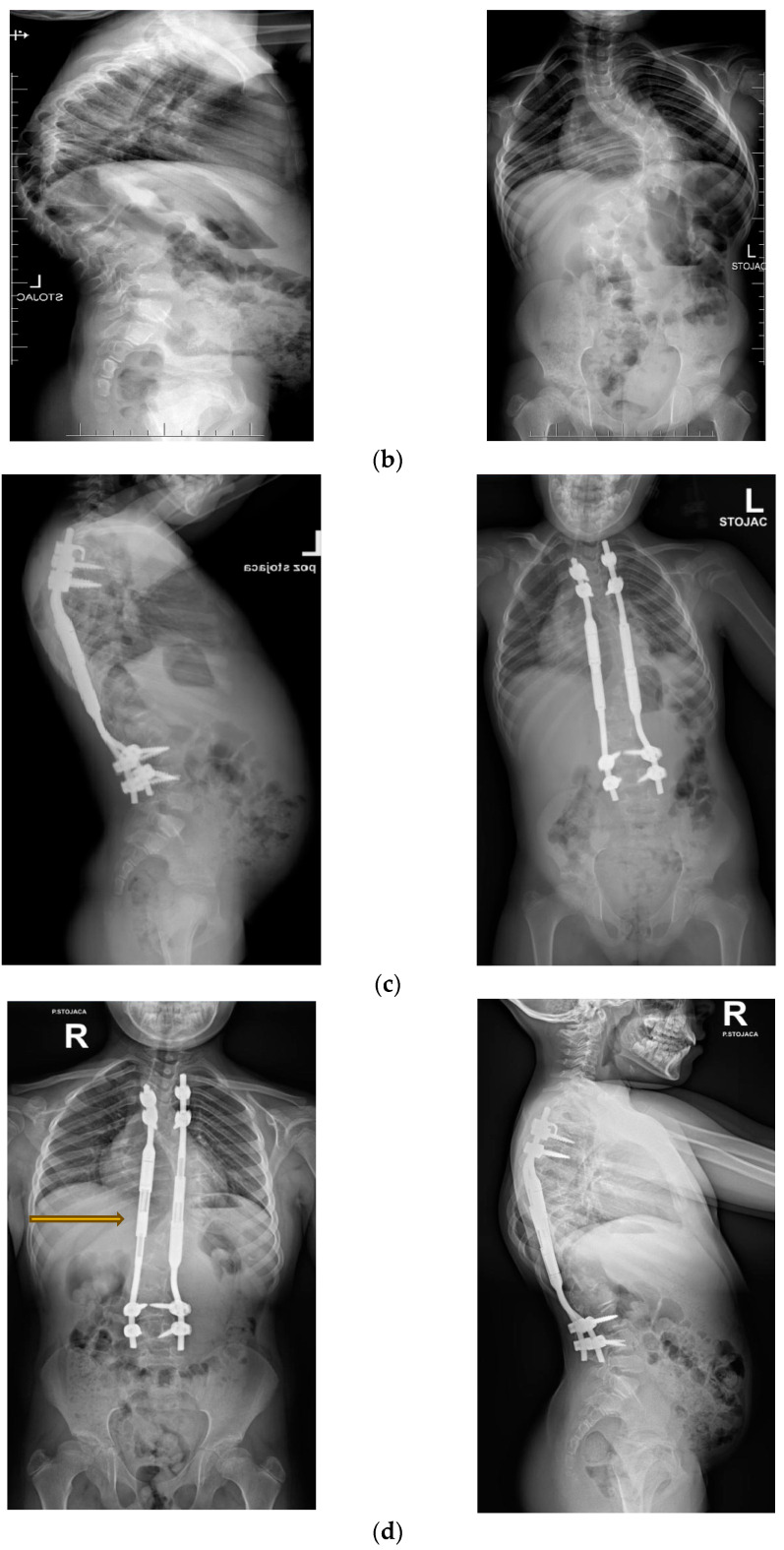

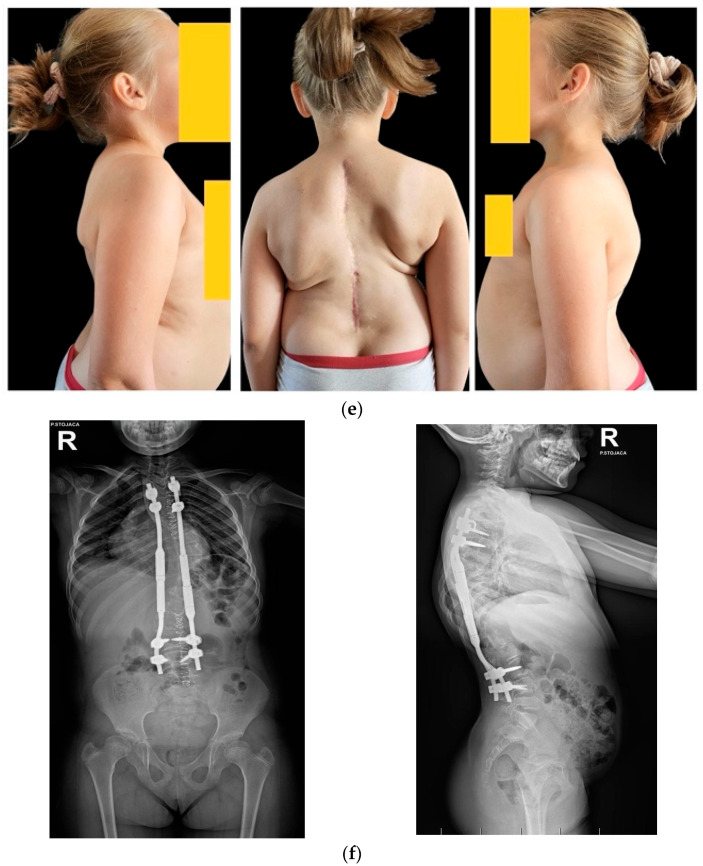
(**a**–**f**) This shows a child with severe kyphoscoliosis (120 degrees of scoliosis, and 105 degrees of thoracic kyphosis) before surgical treatment (**a**,**b**) who underwent initial surgical correction with standard growing rods at 4 years old, who was then treated with MCGRs one year after the initial surgery. (**c**) This case was complicated by a deep infection one month after the conversion, requiring revision surgery. Three years after surgical debridement, the child experienced an MCGR fracture (**d**) as the arrow shows; following this, she underwent revision surgery and MCGR replacement from 4.5 mm to 5.5 mm (**e**,**f**) at the 5-year follow-up.

During the lengthening period, we noted and analyzed the late complications which manifested more than 3 months after the initial procedure. Figure 1 shows a girl with EOS who experienced several complications (an infection, a pin fracture, and two revision surgeries).

**Figure 2:**
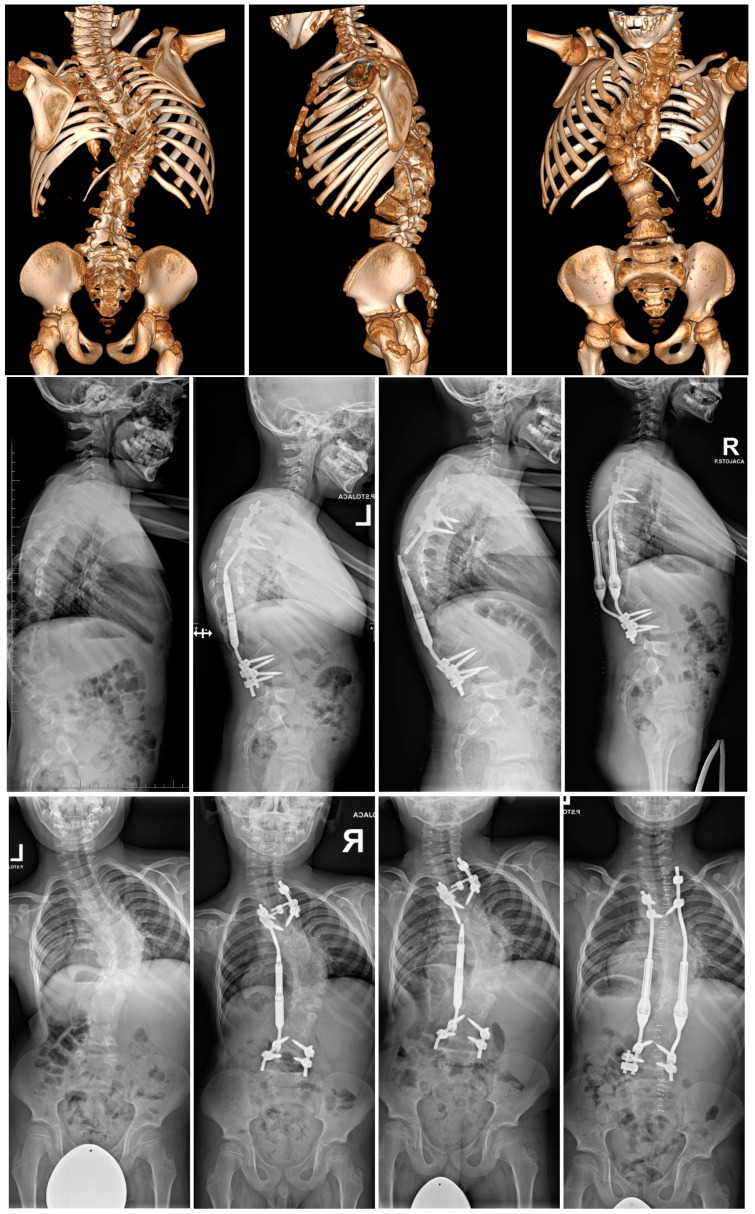
The 6-year-old child with congenital scoliosis treated initially with one MCGR to whom rod fracture occurred at 2-years of the follow-up period. During revision surgery, two minimally invasive controlled growing rods (MICGRs) were used [54].

In this last group, 57 patients developed medical and mechanical complications (35%). The data are depicted in Table 3, with the cohorts divided into those with and without complications. The Figure 2 presents the 6-year-old child with congenital scoliosis treated with one MCGR to whom rod fracture occurred at 2-years of the follow-up period.

Regarding the late complications, 33 patients required unplanned surgery (20%). These complications are presented in detail in Table 4.

**Figure 3:**
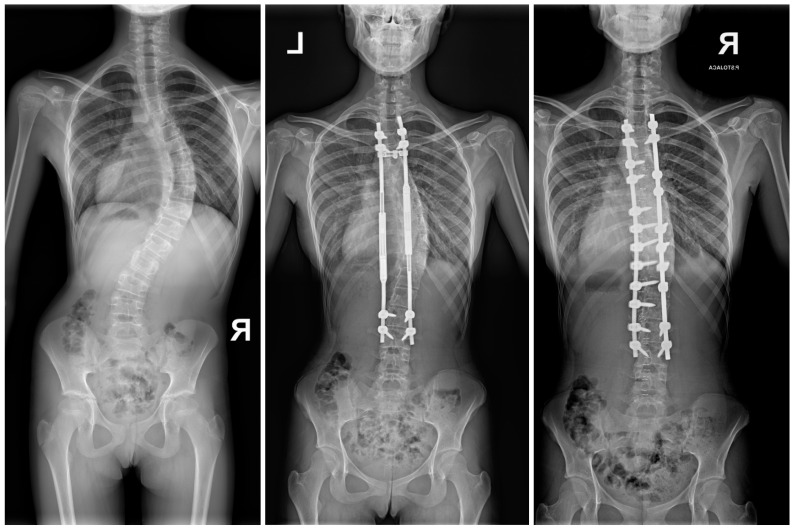

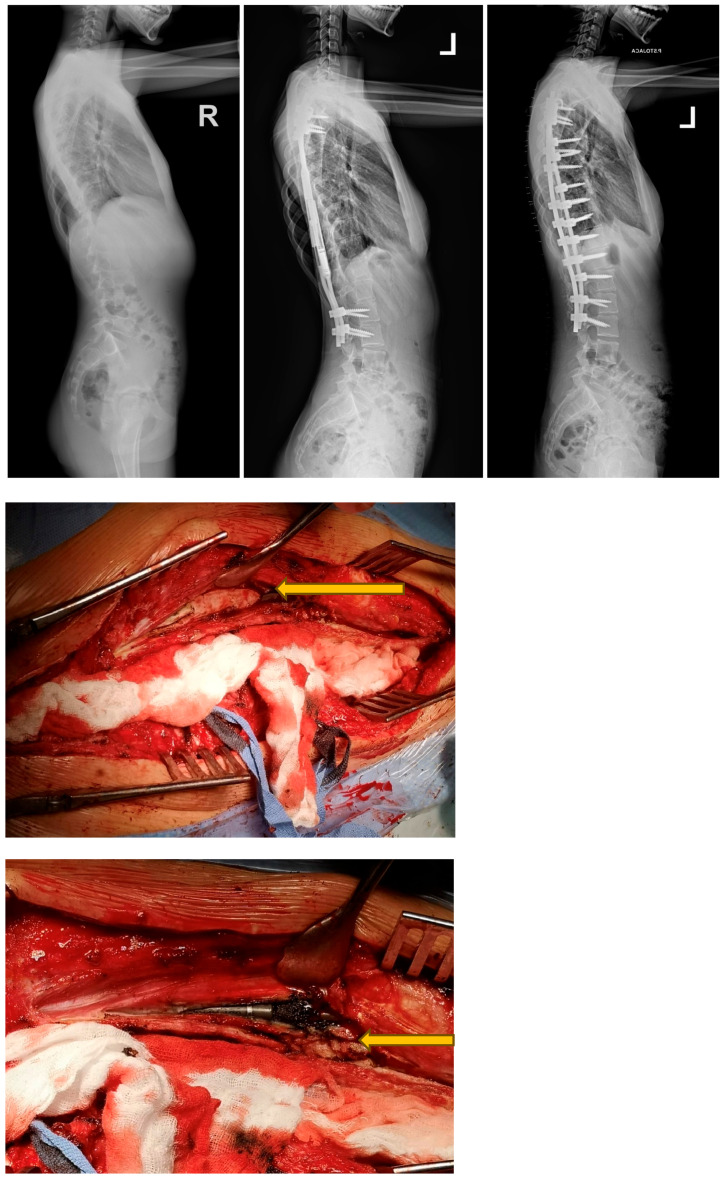

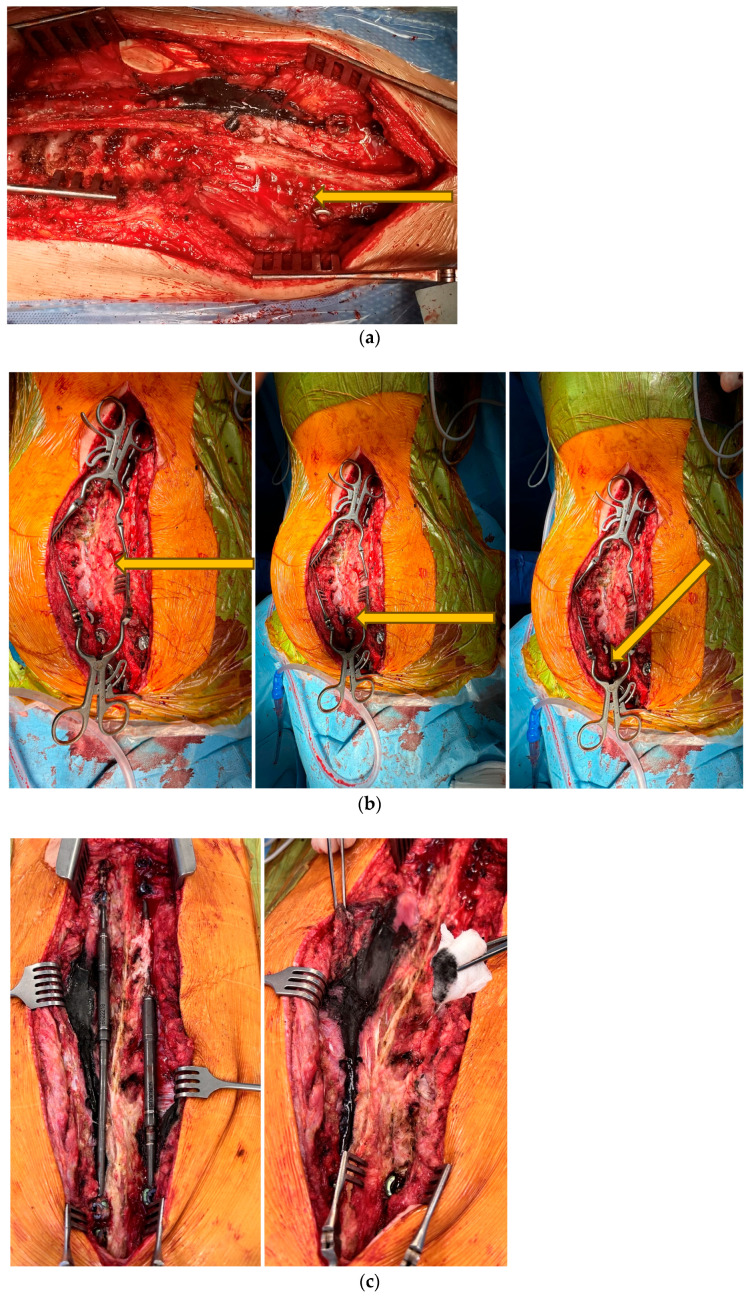
(**a**) X-rays and intraoperative pictures of 8-year-old girl with EOS treated with MCGRs, no complications observed during the 5-years follow-up and lengthening period, but spontaneous fusion, apex ribs fusion, and metallosis observed on the convex side during conversion to PSF, as the arrows show. However, apex ribs fusion is not always particularly visible, so we rely on observations and examination of the correction of the peak of the curve intraoperatively, after removal of both MCGRs. (**b**) Intraoperative pictures of 9-year-old girl with EOS treated with MCGRs, no complications observed during follow-up and lengthening period, but spontaneous fusion, apex ribs fusion, and metallosis observed on the convex side during conversion to PSF, as the arrows show. (**c**) Intraoperative pictures of 12-year-old girl with EOS treated with MCGRs, no complications observed during the follow-up and lengthening period, but spontaneous fusion, apex ribs fusion, and metallosis observed on the convex side during conversion to PSF at 3-years of follow-up period.

After the final fusion, there were no complications or unplanned surgeries among the 48 patients (30%) converted to PSF during the follow-up period, but several specific complications were noted during the revision surgery or conversion to PSF, such as spontaneous vertebrae fusion, rib fusion and metallosis. During surgery, metallosis and spontaneous vertebrae fusion and rib fusion were observed in varying degrees in all the patients converted to PSF. Figure 3a presents X-rays and intraoperative pictures of 8-year-old girl with EOS treated with MCGRs, no complications observed during the 5-year follow-up and lengthening period, but spontaneous fusion, apex ribs fusion and metallosis observed on the convex side. Figure 3b,c present intraoperative pictures of metallosis and spontaneous fusion noted during conversion MCGRs to PSF.

**Figure 4:**
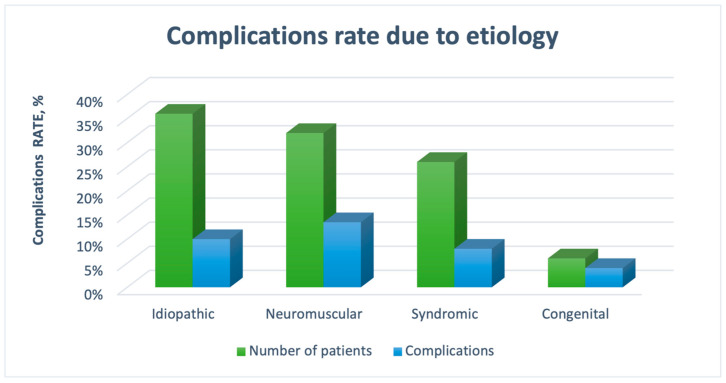
Complication rates of different etiologies.

The comparison of these findings with those of other studies presents challenges due to the absence of the relevant literature. However, based on our observations, spontaneous fusion and metallosis tended to be more pronounced with longer rod placement in the patients. For instance, when the MCGRs were removed two years post-implantation, metallosis and fusion occurred significantly less often than they did five years later. Furthermore, our observations suggest that spontaneous fusion predominantly affects the convex side and becomes more extensive with a prolonged MCGR treatment duration. Figure 4 shows the complication rates of the different etiologies of spinal deformity.

During the total treatment course, we compared the preoperative and postoperative changes in the EOSQ-24 during the follow-up period. Prior to the operation, data from the EOSQ-24 was accessible for analysis in 112 cases. However, upon the final follow-up, data from the EOSQ-24 questionnaire was only accessible for 98 of the initial pool of patients.

**Figure 5:**
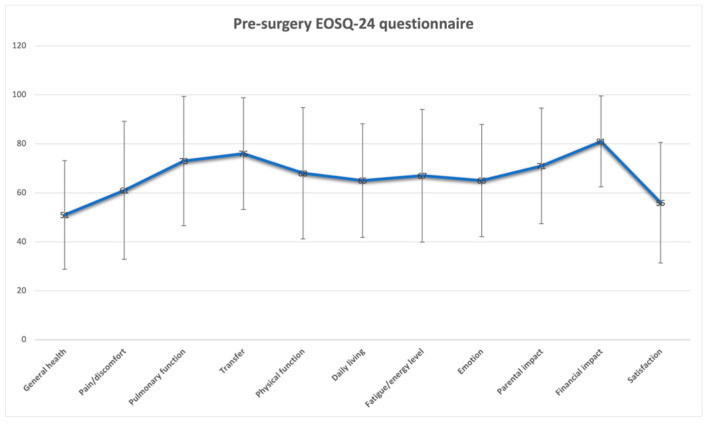
Preoperative EOSQ-24 scores.

**Figure 6:**
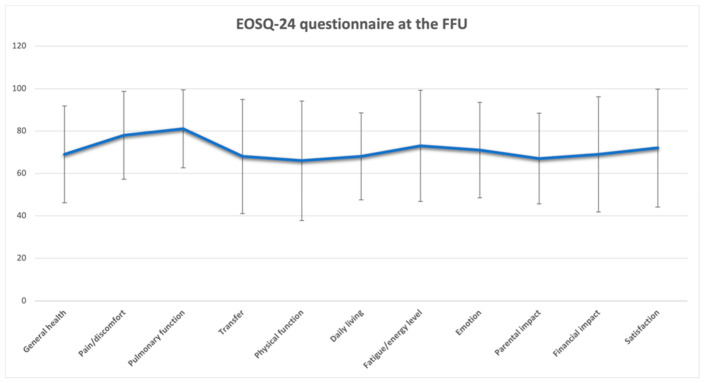
Postoperative EOSQ-24 scores.

Figure 5 and Figure 6 present the preoperative and postoperative EOSQ-24 outcomes, respectively.

**Figure 7:**
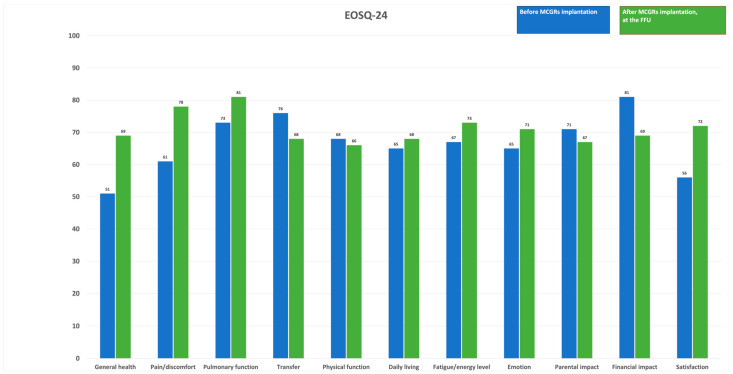
Preoperative EOSQ-24 scores compared to EOSQ-24 values at the final follow-up.

We found significant improvements in the scores for several domains: General Health; Pain; Pulmonary Function; Daily Living; Fatigue and Energy Level; Emotion; and Satisfaction. These could correspond with the reduced number of surgeries, complications, and hospitalizations, but the financial and parental impacts and transfer scores worsened during the follow-up period (as shown in Figure 7).

## 4. Discussion

This multicenter study presents the complications and HRQoL among patients treated with MCGRs. We analyzed the use of magnetic rods to treat early-onset scoliosis in 161 patients. According to the authors’ knowledge, this paper presents data on the complications and quality of life of the largest group of patients with EOS treated with MCGRs. In the previous studies, the radiological outcomes were reported [45,47,55]. TGRs have been the mainstay surgical option for the surgical treatment of EOS [10]. TGRs necessitate frequent manual interventions while under general anesthesia, thereby leading to a heightened likelihood of complications related to anesthesia, implants, alignment, or wounds, whether they be superficial or deep infections; hook or screw dislodgements; rod fractures; prominent implants; junctional kyphosis; curve decompensation; misalignment [3,7,12,20,26,28,31,44]. The introduction of MCGRs aimed to reduce the number of surgeries and, therefore, the number of complications in patients with EOS. MCGRs allow surgeons to perform distractions with a remote-control mechanism without the necessity of surgery, thus reducing the number of surgeries. Repeated surgeries expose the patients to a higher risk of surgery-related infection. MCGRs could potentially lead to a decreased incidence of surgery-related infection [10,56,57].

In the current study, overall, eight patients (classified as intraoperative, early postoperative, and late postoperative complications) developed deep surgical site infection (5%), which resulted in eight reoperations. Kabirian et al. performed a multicenter study that reviewed 379 patients treated with TGRs with a minimum two-year follow-up [28]. They reported deep surgical site infection in 42 patients (11%). Based on our results, the MCGRs resulted in a lower infection rate (5% for the MCGRs vs. 11% for the TGRs). Choi et al. [17] also reported a lower infection rate in the patients treated with MCGRs vs. TGRs (4% for the MCGRs vs. 11% for the TGRs). A similar infection rate related to MCGRs was reported by Urbański et al.: 4.2% [56].

Bess et al. [3] documented that 58% of the 140 patients subjected to TGRs experienced complications. The researchers noted a correlation between the number of surgical procedures and the incidence of complications. In their cohort, the complication rate stood at 40% following two procedures, escalating to 100% for patients who underwent more than eleven procedures. Additionally, they observed a heightened risk of wound complications with each subsequent surgery. The likelihood of facing a complication rose by 24% with every additional surgical procedure beyond the initial operation [3].

This could explain the lower incidence of complications in the patients with MCGRs who undergo fewer surgeries. The overall late complication rate in our study group was 35% (57 patients). In addition, there were complications related to implants; in nine patients (5.7%), the screws pulled out, which was associated with two unplanned reoperations, while in seven patients (4.4%), the screws or hooks loosened. A rod slippage failure was observed in 33 patients (20.5%), which resulted in unplanned reoperations in 11 cases (6.8%), and rod fracture in 6 patients (3.7%), which was associated with reoperations. The worsening of spinal deformation by more than 10 degrees occurred in six patients, i.e., 3.7%, which resulted in two reoperations, and PJK occurred in eight patients, i.e., in 11.2%, which resulted in another three reoperations. Some other complications included twenty-one cases of implant prominence, one case of subcutaneous pneumothorax, and transient radiculopathy in five patients, which did not require additional procedures. A total of 33 reoperations were performed, which represents 20.5%.

In all the patients converted to PSF (48 patients, 30%), metallosis was observed (Figure 3a–c). Moreover, it was found in 70% of children who underwent revision, i.e., in 23 patients, which, in our opinion, is related to the long implant retention period and the telescopic movement phenomenon. A significant percentage of the metallosis cases is consistent with the report by Zhang et al. [50]. Metallosis, a phenomenon characterized by the presence of metallic debris in the tissues, can be elucidated through two primary mechanisms. Firstly, growth marks observed on the elongating bar of the rod may indicate the occurrence of high stress levels during the lengthening process. Alternatively, metallosis can also be attributed to the escape of titanium particles from the internal components of the rod mechanism, as evidenced in previous research findings [10,57]. Within the scope of our investigation, metallosis manifested most prominently in the vicinity of the moving components of the medical device, suggesting a potential correlation between the location of metallosis and the functionality of the device.

Akbarnia et al. [10] compared the complication rate of MCGRs versus TGRs for the treatment of early-onset scoliosis. The authors presented a group of 17 patients treated with MCGRs, of which 12 patients underwent complete analysis; they were observed for 2 years. For each of the 12 patients, there was a corresponding patient treated with TGRs. Among four MCGR patients (33%) four medical- and eight implant-related issues occurred, with the latter four causing reoperation; these involved two screw extractions, one case of implant prominence, and one loss of correction. However, eleven TGR patients (95%) experienced four infections, eight medical complications, and thirteen implant-related complications, which required two wound debridements in one patient, three implant revisions in two patients, and ten surgical extensions due to implant-related complications. Urbański et al. [56] in a group of 47 EOS patients treated with MCGRs, described 17 complications in 16 patients (34%). Complications related to instrumentation were observed in eight patients (17%), resulting in eight additional procedures. Two patients underwent reoperation due to a deep wound infection (4.2%). In five patients (10.6%), there were problems with extending the magnetic rod, which resulted in three reoperations. PJK was observed in one patient, who did not require an additional surgery. The authors observed more complications in the patients with one rod than they did among those with two rods, 45.8% vs. 30.4%, respectively. A total of 13 additional surgeries were performed. Attention has been drawn to the high frequency of complications in patients with syndromic scoliosis, which is consistent with the results obtained in our patients [58,59,60,61]. The higher incidence of complications with a one-rod TGR vs. double TGR construct has been reported by other authors [3]. The higher incidence of complications related to single-rod MCRRs is not necessarily related to the type of implant used, but to the fact that only one rod is used. Hellenius et al. [17] determined an increasing rate of complications in the patients treated with magnetic rods related to the severity of curvature before treatment, amounting to 88%, with a Cobb angle above 90 degrees. This is consistent with our observation that significantly more complications occur in patients with scoliosis that exceeds 90-degree Cobb angle than among those with scoliosis under 90 degrees, 23% vs. 8% of all the patients, respectively. Some authors have described the implant prominence of MCGRs [16,34,37,58,62,63]. Implant prominence was observed in 13% of the patients of our cohort (21 pts), but no patient required reoperation for this reason.

In our study, we compared the preoperative and 2-year postoperative changes in the EOSQ-24. We found significant improvements in scores for three domains: General Health; Pain Pulmonary Function; Daily Living; Fatigue and Energy Level; Emotion; and Satisfaction. These findings could correspond with the reduced number of surgeries, complications, and hospitalizations [64]. The financial and parental impacts and transfer scores worsened during the follow-up period (as shown in Figure 5). [29,52,53]. These alterations are deemed to be of significance in clinical settings as they exceed the minimum clinically important difference (MCID) when utilizing distribution-based methodologies (for instance, a variation of more than 1 standard error of measurement or over 0.2 standard deviations) [29,65]. Nevertheless, there is a lack of established formal MCIDs for the EOSQ-24 at present, leading to preliminary interpretations regarding the clinical significance of the findings. It is important to note that not all EOSQ-24 domain scores exhibited enhancements as time progressed, particularly evident in the lack of improvement in the Parental Impact, Financial Impact, Physical Function, and Transfer domains. Matsumoto et al. [31] compared the preoperative and postoperative HRQoL scores for patients with MCGRs vs. TGRs. By comparing the 2-year postoperative and preoperative assessments of the patients with MCGRs, the results demonstrate significant improvement in four domains (HRQoL, Parental Burden, Financial Burden, and Satisfaction). In the HRQoL domain, three of the eight subdomains showed statistically significant improvements (Physical Function, Daily Living, and Emotion), and four sub-domains tended toward significant improvements (General Health, Pain/Discomfort, Pulmonary Function, and Transfer). The Fatigue/Energy Level slightly decreased at the 2-year postoperative assessment, by 1.6 points. By comparing the 2-year follow-up results for the MCGR vs. TGR groups, the MCGR group showed a greater increase in scores in Pain, Children Satisfaction, and Parent Satisfaction, but without statistical significance. Oral et al. [32] compared the ESOQ-24 pre-surgery and final follow-up results (14.9 months on average). The final follow-up revealed a significant improvement in the quality-of-life scores (*p* < 0.05). This improvement was evident when assessing the EOSQ-24 scores across various categories. Notably, there were no notable differences between the pre- and postoperative scores in areas such as General Health, Pain and Discomfort, Respiratory Function, Movement Capability, Physical Function, or Family Life (*p* > 0.05). However, there was a substantial improvement in scores related to Exhaustion and Energy Levels, Emotional Status, the impact of the disease on both parents and patients, as well as parent satisfaction (*p* < 0.05) at the final follow-up. Moreover, the scores reflecting Financial Effects showed a decrease at the final follow-up (*p* < 0.05).

### Limitations

The limitation of our study is its retrospective nature. The consistency of the data reported and analyzed may have varied among the study sites. The patients were operated on by several surgeons, and although the treatment method was the same, the surgical technique may have differed between the surgeons. The utilization of MCGRs represents a quite novel surgical approach, and within a limited timeframe following their implementation, patients may undergo a transition to PSF (as evidenced by 30% of patients in our study). Therefore, it is conceivable that we have witnessed a process of acquisition of knowledge. Another limitation of this study is the lack of data on the patients’ respiratory function. One of the strengths of our research lies in the extensive number of patients included and the prolonged duration of follow-up. The initiation of MCGR treatment persisted until the conclusion of PSF, constituting one of the most sizable cohorts when juxtaposed with the existing literature. Prior to the operation, data from the EOSQ-24 questionnaire was accessible for analysis in 112 cases. However, upon the final follow-up, data from the EOSQ-24 questionnaire was only accessible for 98 of the initial pool of patients. This reduction in the number of available data sets might be attributed to various factors such as dropouts from the study, incomplete responses, or other unforeseen circumstances. It is imperative to consider these limitations when interpreting the results of the study and making any conclusions based on the data collected. Future research endeavors in this field should aim to address these limitations and strive for a more comprehensive collection of data to ensure a more robust and reliable analysis of the HRQoL in children with EOS and their families. A notable restriction in our research is the absence of a control cohort comprising patients who underwent treatment with conventional growing rods; this would have enabled a direct comparison of treatment outcomes with patients undergoing treatment with magnetically controlled growing rods, thus providing a more comprehensive analysis of the efficacy and safety of the latter.

## 5. Conclusions

The management of early-onset scoliosis with MCGR treatment poses a general risk of complications, leading to unforeseen surgical procedures in around 26% of patients throughout the treatment process. The total number of complications during the entire treatment course is estimated at 66%. This study identified the factors contributing to an increasing rate of complications in patients treated with MCGRs: neuromuscular etiology, a curvature greater than 90 degrees, being female, age less than 6 years old, and the rod diameter 5.0 mm and 4.5 mm. Deformity correction and spine growth showed similar results in cases treated with either single or double rods. The use of MCGRs helps in reducing the necessity for multiple elective surgeries to lengthen the instrumentation.

## Figures and Tables

**Table 1:** The intraoperative complications, n.

Complications	N = 161 Pts, (%)	Action
Pneumothorax due to intrathecal catheter	5 (3.1%)	Conservative treatment/full recovery within 8 days of surgery
Horner syndrome	2 (1.3%)	Conservative treatment/full recovery within 21 days of surgery
Dural tear	3 (1.9%)	Full recovery after revision surgery
Deep wound infection	2 (1.3%)	Full recovery after revision surgery
Superficial wound infection	3 (1.9%)	Conservative treatment/full recovery within 30 days of surgery
Pull-out upper construct	2 (1.3%)	Full recovery after revision surgery
Transient neurological deficit	2 (1.3%)	Conservative treatment/full recovery within 14 days of surgery
Radiculopathy	2 (1.3%)	Conservative treatment/full recovery within 21 days of surgery
Irritation of the lateral thigh cutaneous nerve	6 (3.7%)	Conservative treatment/full recovery within 30 days of surgery
Superior Mesenteric Artery Syndrome (SMAS)	2 (1.3%)	Conservative treatment/full recovery within 14 days of surgery
Pneumonia	4 (2.5%)	Conservative treatment/full recovery within 14 days of surgery
TOTAL	33 (20.5%)	7 (4.3%) revision surgeries

**Table 2:** The early postoperative complications, n.

Complications	N = 161 Pts, (%)	Action
Deep wound infection	2 (1.3%)	Full recovery after revision surgery
Superficial wound infection	3 (1.9%)	Conservative treatment
Radiculopathy	2 (1.3%)	Conservative treatment/full recovery after physiotherapy
Back pain	8 (5%)	Conservative treatment/physiotherapy
Subcutaneous pneumothorax	1 (0.6%)	Conservative treatment
TOTAL	16 (10%)	2 (1.3%) revision surgeries

**Table 3:** Cohorts of patients with and without late? complications during lengthening treatment.

Demographic/Complications n = 161	A Number of Patients with at Least One Late Complication n = 57	B Number of Patients with No Late Complications n = 104	*p*-Value A vs. B
N (%)	57 (35%)	104 (65%)	
Age in years (SD) at insertion of MCGR (range)	6.8 (2.9)	6.42 (2.03)	N. S.
	(2.5–13.5)	(3.5–11)	
Mean (SD) FU, months			
(range)	37.5 (24.5)	31.2 (26.2)	N. S.
	(12–68)	(12–68)	
Gender, n (%)			
Male	14 (9%)	57 (35%)	N. S.
Female *p*-Value (a. vs. b.)	43 (27%) *p* < 0.001	47 (29%) N. S.	N. S.
Etiology, n (%)			
Congenital	7 (4%)	4 (2.5%)	N. S.
Idiopathic	16 (10%)	42 (26%)	N. S.
Neuromuscular	21 (13%)	29 (18%)	N. S.
Syndromic	13 (8%)	29 (18%)	N. S.
Rod diameter, n (%) of pts			
a. 4.5 mm, 5.0 mm	32 (20%)	22 (14%)	N. S.
b. 5.5 mm, 6.0 mm	20 (12%)	87 (54%)	N. S.
*p*-Value (a. vs. b.)	*p* < 0.05	N. S.	
Rods length, n (%) of pts			
a. 70 mm	34 (21%)	35 (22%)	
b. 90 mm	18 (11%)	74 (46%)	
*p*-Value (a. vs. b.)	*p* < 0.001	N. S.	
Patients’ age, n (%)			
a. less than 6 y.o	35 (22%)	38 (23%)	N. S.
b. more than 6 y.o	17 (11%)	71 (44%)	N. S.
*p*-Value (a. vs. b.)	*p* < 0.001	N. S.	
MC, n (%)			
a. more than 90 degrees	38 (23%)	28 (19%)	N. S.
b. less than 90 degrees	14 (8%)	81 (50%)	N. S.
*p*-Value (a. vs. b.)	*p* < 0.001	N. S.	

N.S. means not significant

**Table 4:** Postoperative late complications (during lengthening).

Complications	IS (n = 58)	SS (n = 42)	NS (n = 51)	CS (n = 10)	Total Value
Infection					
Superficial	1	0	1	1	3
Deep	0	1	3	0	4
Total infection rate	1	1	4	1	7 (4.3%)
Anchor pull-out					
Upper	2	2	0	0	4
Lower	0	0	3	1	4
Total anchor pull-out	2	2	3	1	8 (5%)
Rod breakage	4	2	4	1	11 (6.8%)
Pin fracture	1	2	3	0	6 (3.8%)
Distraction failure	7	3	5	3	18 (12%)
Adding on	0	1	0	0	1 (0.6%)
PJK	1	2	2	1	6 (3.8%)
Total	16 (10%)	13 (8%)	21 (13%)	7 (4%)	57 (35%)

## Data Availability

The data are contained within the article.

## References

[1] Helenius I.J. (2020). Standard and magnetically controlled growing rods for the treatment of early onset scoliosis. Ann. Transl. Med..

[2] Karol L.A. (2019). The Natural History of Early-onset Scoliosis. J. Pediatr. Orthop..

[3] Bess S., Akbarnia B.A., Thompson G.H., Sponseller P.D., Shah S.A., El Sebaie H., Boachie-Adjei O., Karlin L.I., Canale S., Poe-Kochert C. (2010). Complications of growing-rod treatment for early-onset scoliosis: Analysis of one hundred and forty patients. J. Bone Jt. Surg. Am..

[4] Danielewicz A., Wójciak M., Sawicki J., Dresler S., Sowa I., Latalski M. (2021). Comparison of Different Surgical Systems for Treatment of Early-Onset Scoliosis in the Context of Release of Titanium Ions. Spine.

[5] Latalski M., Starobrat G., Fatyga M., Sowa I., Wójciak M., Wessely-Szponder J., Dresler S., Danielewicz A. (2022). Wound-Related Complication in Growth-Friendly Spinal Surgeries for Early-Onset Scoliosis-Literature Review. J. Clin. Med..

[6] Bednar E.D., Bergin B., Kishta W. (2021). Comparison of Magnetically Controlled Growing Rods with Other Distraction-Based Surgical Technologies for Early-Onset Scoliosis: A Systematic Review and Meta-Analysis. JBJS Rev..

[7] Varley E.S., Pawelek J.B., Mundis G.M., Oetgen M.E., Sturm P.F., Akbarnia B.A., Yaszay B. (2021). The role of traditional growing rods in the era of magnetically controlled growing rods for the treatment of early-onset scoliosis. Spine Deform..

[8] Saarinen A.J., Sponseller P.D., Andras L.M., Skaggs D.L., Emans J.B., Thompson G.H., Helenius I.J., Pediatric Spine Study Group (2022). Matched comparison of magnetically controlled growing rods with traditional growing rods in severe early-onset scoliosis of ≥90°: An interim report on outcomes 2 years after treatment. J. Bone Jt. Surg. Am..

[9] Wijdicks S.P.J., Tromp I.N., Yazici M., Kempen D.H.R., Castelein R.M., Kruyt M.C. (2019). A comparison of growth among growth-friendly systems for scoliosis: A systematic review. Spine J..

[10] Akbarnia B.A., Pawelek J.B., Cheung K.M., Demirkiran G., Elsebaie H., Emans J.B., Johnston C.E., Mundis G.M., Noordeen H., Skaggs D.L. (2014). Traditional growing rods versus magnetically controlled growing rods for the surgical treatment of early-onset scoliosis: A case-matched 2-year study. Spine Deform..

[11] Migliorini F., Chiu W.O., Scrofani R., Chiu W.K., Baroncini A., Iaconetta G., Maffulli N. (2022). Magnetically controlled growing rods in the management of early onset scoliosis: A systematic review. J. Orthop. Surg. Res..

[12] Kim G., Sammak S.E., Michalopoulos G.D., Mualem W., Pinter Z.W., Freedman B.A., Bydon M. (2022). Comparison of surgical interventions for the treatment of early-onset scoliosis: A systematic review and meta-analysis. J. Neurosurg. Pediatr..

[13] Tognini M., Hothi H., Dal Gal E., Shafafy M., Nnadi C., Tucker S., Henckel J., Hart A. (2021). Understanding the implant performance of magnetically controlled growing spine rods: A review article. Eur. Spine J..

[14] Xu G.J., Fu X., Tian P., Ma J.X., Ma X.L. (2016). Comparison of single and dual growing rods in the treatment of early onset scoliosis: A meta-analysis. J. Orthop. Surg. Res..

[15] Flynn J.M., Tomlinson L.A., Pawelek J., Thompson G.H., McCarthy R., Akbarnia B.A. (2013). Growing Spine Study Group. Growing-rod graduates: Lessons learned from ninety-nine patients who completed lengthening. J. Bone Jt. Surg. Am..

[16] Cheung J.P.Y., Yiu K., Kwan K., Cheung K.M.C. (2019). Mean 6-year follow-up of magnetically controlled growing rod patients with early onset scoliosis: A glimpse of what happens to graduates. Neurosurgery.

[17] Helenius I.J., Sponseller P.D., McClung A., Pawelek J.B., Yazici M., Emans J.B., Thompson G.H., Johnston C.E., Shah S.A., Akbarnia B.A. (2019). Surgical and Health-related Quality-of-Life Outcomes of Growing Rod “Graduates” with Severe versus Moderate Early-onset Scoliosis. Spine.

[18] Bekmez S., Dede O., Yazici M. (2017). Advances in growing rods treatment for early onset scoliosis. Curr. Opin. Pediatr..

[19] Mackey C., Hanstein R., Lo Y., Vaughan M., St Hilaire T., Luhmann S.J., Vitale M.G., Glotzbecker M.P., Samdani A., Parent S. (2022). Magnetically Controlled Growing Rods (MCGR) Versus Single Posterior Spinal Fusion (PSF) Versus Vertebral Body Tether (VBT) in Older Early Onset Scoliosis (EOS) Patients: How Do Early Outcomes Compare?. Spine.

[20] Akbarnia B.A., Emans J.B. (2010). Complications of Growth-Sparing Surgery in Early Onset Scoliosis. Spine.

[21] Keskinen H., Helenius I., Nnadi C., Cheung K., Ferguson J., Mundis G., Pawelek J., Akbarnia B.A. (2016). Preliminary comparison of primary and conversion surgery with magnetically controlled growing rods in children with early onset scoliosis. Eur. Spine J..

[22] McIntosh A.L., Booth A., Oetgen M.E. (2024). Unplanned return to the operating room (UPROR) occurs in 40% of MCGR patients at an average of 2 years after initial implantation. Spine Deform..

[23] Jamnik A.A., Shaw K.A., Thornberg D., McClung A., Jo C.H., Ramo B., McIntosh A. (2024). Health-related quality of life and clinical outcomes for magnetically controlled growing rod patients after treatment termination. Spine Deform..

[24] Menapace B., Jain V., Sturm P. (2024). Autofusion in early-onset scoliosis growing constructs: Occurrence, risk factors, and impacts. Spine Deform..

[25] Yang M.J., Rompala A., Samuel S.P., Samdani A., Pahys J., Hwang S. (2023). Autofusion with Magnetically Controlled Growing Rods: A Case Report. Cureus.

[26] Yang J.S., Sponseller P.D., Thompson G.H., Akbarnia B.A., Emans J.B., Yazici M., Skaggs D.L., Shah S.A., Salari P., Poe-Kochert C. (2011). Growing Spine Study Group. Growing rod fractures: Risk factors and opportunities for prevention. Spine.

[27] Thakar C., Kieser D.C., Mardare M., Haleem S., Fairbank J., Nnadi C. (2018). Systematic review of the complications associated with magnetically controlled growing rods for the treatment of early onset scoliosis. Eur. Spine J..

[28] Kabirian N., Akbarnia B.A., Pawelek J.B., Alam M., Mundis G.M., Acacio R., Thompson G.H., Marks D.S., Gardner A., FRCS (2014). Deep surgical site infection following 2344 growing-rod procedures for early-onset scoliosis: Risk factors and clinical consequences. J. Bone Jt. Surg..

[29] Lemans J.V.C., Top A., Tabeling C.S., Scholten E.P., Stempels H.W., Schlösser T.P.C., Castelein R.M., Kruyt M.C. (2024). Health-related quality of life in early onset scoliosis patients treated with the spring distraction system: What to expect in the first 2 years after surgery. Spine Deform..

[30] Corona J., Matsumoto H., Roye D.P., Vitale M.G. (2011). Measuring quality of life in children with early onset scoliosis: Development and initial validation of the early onset scoliosis questionnaire. J. Pediatr. Orthop..

[31] Matsumoto H., Skaggs D.L., Akbarnia B.A., Pawelek J.B., Hilaire T.S., Levine S., Sturm P., Perez-Grueso F.J.S., Luhmann S.J., Sponseller P.D. (2021). Comparing health-related quality of life and burden of care between early-onset scoliosis patients treated with magnetically controlled growing rods and traditional growing rods: A multicenter study. Spine Deform..

[32] Oral I., Sahin Y., Mert M., Oner A., Kargin D., Albayrak A., Balioglu M.B., Kaygusuz M.A. (2021). Quality of Life Among Patients with Early-Onset Scoliosis Treated with Magnetically Controlled Growing Rods—Early-Term Results. World Neurosurg..

[33] Hatem A., Elmorshidy E.M., Elkot A., Hassan K.M., El-Sharkawi M. (2024). Autofusion in growing rod surgery for early onset scoliosis; what do we know so far?. SICOT J..

[34] Nematian H., Clarke A., Hedayat E., Vahdati Z., Milan N., Mehrpour S.R., Nabian M.H., Mazda K. (2022). Complications of single growing rod constructs in the treatment of severe early-onset scoliosis: A lesson relearned. Spine Deform..

[35] Cheung P.W.H., Wong C.K.H., Sadiang-Abay J.T., Lau S.T., Cheung J.P.Y. (2022). Longitudinal comparison of direct medical cost, radiological and health-related quality of life treatment outcomes between traditional growing rods and magnetically controlled growing rods from preoperative to maturity. BMC Musculoskelet. Disord..

[36] Williams B.A., Matsumoto H., McCalla D.J., Akbarnia B.A., Blakemore L.C., Betz R.R., Flynn J.M., Johnston C.E., McCarthy R.E., Roye D.P. (2014). Development and initial validation of the Classification of Early-Onset Scoliosis (C-EOS). J. Bone Jt. Surg. Am..

[37] Cheung K.M., Cheung J.P., Samartzis D., Mak K.C., Wong Y.W., Cheung W.Y., Akbarnia B.A., Luk K.D. (2012). Magnetically controlled growing rods for severe spinal curvature in young children: A prospective case series. Lancet.

[38] Ilharreborde B., Simon A.L., Shadi M., Kotwicki T. (2023). Is scoliosis a source of pain?. J. Child. Orthop..

[39] Cheung J.P., Yiu K.K., Samartzis D., Kwan K., Tan B.B., Cheung K.M. (2018). Rod lengthening with the magnetically controlled growing rod: Factors influencing rod slippage and reduced gains during distractions. Spine.

[40] Farshad M., Aichmair A., Gerber C., Bauer D.E. (2020). Classification of perioperative complications in spine surgery. Spine J..

[41] Teoh K.H., von Ruhland C., Evans S.L., James S.H., Jones A., Howes J., Davies P.R., Ahuja S. (2016). Metallosis following implantation of magnetically controlled growing rods in the treatment of scoliosis: A case series. Bone Jt. J..

[42] Landriel Ibañez F.A., Hem S., Ajler P., Vecchi E., Ciraolo C., Baccanelli M., Tramontano R., Knezevich F., Carrizo A. (2011). A new classification of complications in neurosurgery. World Neurosurg..

[43] Ridolfi D., Oyekan A.A., Tang M.Y., Chen S.R., Como C.J., Dalton J., Gannon E.J., Jackson K.L., Bible J.E., Kowalski C. (2024). Modified Clavien-Dindo-Sink Classification System for operative complications in adult spine surgery. J. Neurosurg. Spine..

[44] Smith J.T., Johnston C., Skaggs D., Flynn J., Vitale M. (2015). A New Classification System to Report Complications in Growing Spine Surgery: A Multicenter Consensus Study. J. Pediatr. Orthop..

[45] Grabala P., Gupta M.C., Pereira D.E., Latalski M., Danielewicz A., Glowka P., Grabala M. (2024). Radiological Outcomes of Magnetically Controlled Growing Rods for the Treatment of Children with Various Etiologies of Early-Onset Scoliosis—A Multicenter Study. J. Clin. Med..

[46] Daroszewski P., Huber J., Kaczmarek K., Janusz P., Główka P., Tomaszewski M., Kotwicki T. (2024). "Real-Time Neuromonitoring" Increases the Safety and Non-Invasiveness and Shortens the Duration of Idiopathic Scoliosis Surgery. J. Clin. Med..

[47] Grabala P., Gupta M.C., Pereira D.E., Latalski M., Danielewicz A., Glowka P., Grabala M. (2024). Reply to Tabeling et al. Comment on “Grabala et al. Radiological Outcomes of Magnetically Controlled Growing Rods for the Treatment of Children with Various Etiologies of Early-Onset Scoliosis-A Multicenter Study. *J. Clin. Med.*
**2024**, *13*, 1529”. J. Clin. Med..

[48] Cheung J.P.Y., Bow C., Samartzis D., Kwan K., Cheung K.M.C. (2016). Frequent Small Distractions with a Magnetically Controlled Growing Rod for Early-Onset Scoliosis and Avoidance of the Law of Diminishing Returns. J. Orthop. Surg..

[49] Cheung J.P.Y., Bow C., Cheung K.M.C. (2020). “Law of Temporary Diminishing Distraction Gains”: The Phenomenon of Temporary Diminished Distraction Lengths with Magnetically Controlled Growing Rods That Is Reverted with Rod Exchange. Glob. Spine J..

[50] Zhang T., Sze K.Y., Peng Z.W., Cheung K.M.C., Lui Y.F., Wong Y.W., Lui Y.F., Wong Y.W., Kwan K.Y.H., Cheung J.P.Y. (2020). Systematic investigation of metallosis associated with magnetically controlled growing rod implantation for early-onset scoliosis. Bone Jt. J..

[51] Agarwal A., Kelkar A., Agarwal A.G., Jayaswal D., Jayaswal A., Shendge V. (2019). Device-related complications associated with Magec rod usage for distraction-based correction of scoliosis. Spine Surg. Relat. Res..

[52] Matsumoto H., Williams B., Park H.Y., Yoshimachi J.Y., Roye B.D., Roye D.P., Akbarnia B.A., Emans J., Skaggs D., Smith J.T. (2018). The Final 24-Item Early Onset Scoliosis Questionnaires (EOSQ-24): Validity, Reliability and Responsiveness. J. Pediatr. Orthop..

[53] Wijdicks S.P.J., Dompeling S.D., de Reuver S., Kempen D.H.R., Castelein R.M., Kruyt M.C. (2019). Reliability and Validity of the Adapted Dutch Version of the Early-Onset Scoliosis-24-Item Questionnaire (EOSQ-24). Spine.

[54] Grabala P. (2024). Minimally Invasive Controlled Growing Rods for the Surgical Treatment of Early-Onset Scoliosis—A Surgical Technique Video. J. Pers. Med..

[55] Tabeling C.S., Lemans J.V.C., Kruyt M.C. (2024). Comment on Grabala et al. Radiological Outcomes of Magnetically Controlled Growing Rods for the Treatment of Children with Various Etiologies of Early-Onset Scoliosis—A Multicenter Study. *J. Clin. Med.*
**2024**, *13*, 1529. J. Clin. Med..

[56] Urbański W., Tucker T., Ember T., Nadarajah R. (2020). Single vs. dual rod constructs in early onset treated with magnetically controlled growing rods. Adv. Clin. Exp. Med..

[57] Joyce T.J., Smith S.L., Rushton P.R.P., Bowey A.J., Gibson M.J. (2018). Analysis of explanted magnetically controlled growing rods from seven UK spinal centers. Spine.

[58] Yokogawa N., Demura S., Ohara T., Tauchi R., Takimura K., Yanagida H., Yamaguchi T., Watanabe K., Suzuki S., Uno K. (2024). Instrumentation failure following pediatric spine deformity growth-sparing surgery using traditional growing rods or vertical expandable prosthetic titanium ribs. BMC Musculoskelet. Disord..

[59] Hariharan A.R., Shah S.A., Sponseller P.D., Yaszay B., Glotzbecker M.P., Thompson G.H., Cahill P.J., Bastrom T.P., Pediatric Spine Study Group, Harms Study Group (2023). Definitive fusions are better than growing rod procedures for juvenile patients with cerebral palsy and scoliosis: A prospective comparative cohort study. Spine Deform..

[60] Shah S.A., Karatas A.F., Dhawale A.A., Dede O., Mundis G.M., Holmes L., Yorgova P., Neiss G., Johnston C.E., Emans J.B. (2014). The effect of serial growing rod lengthening on the sagittal profile and pelvic parameters in early-onset scoliosis. Spine.

[61] Ilharreborde B., Ponchelet L., Sales de Gauzy J., Choufani E., Baudoux M., Pesenti S., Simon A.L. (2022). How does magnetically controlled growing rods insertion affect sagittal alignment in ambulatory early onset scoliosis patients?. Eur. Spine J..

[62] Choi E., Yaszay B., Mundis G., Hosseini P., Pawelek J., Alanay A., Berk H., Cheung K., Demirkiran G., Ferguson J. (2017). Implant Complications After Magnetically Controlled Growing Rods for Early Onset Scoliosis. J. Pediatr. Orthop..

[63] Heydar A.M., Şirazi S., Bezer M. (2016). Magnetic Controlled Growing Rods as a Treatment of Early Onset Scoliosis: Early Results with Two Patients. Spine.

[64] Dahl B., Dragsted C., Ohrt-Nissen S., Andersen T., Gehrchen M. (2018). Use of a distraction-to-stall lengthening procedure in magnetically controlled growing rods: A single-center cohort study. J. Orthop. Surg..

[65] Copay A.G., Subach B.R., Glassman S.D., Polly D.W., Schuler T.C. (2007). Understanding the minimum clinically important difference: A review of concepts and methods. Spine J..

